# Influence of the Conditioning Method for Pre-Sintered Zirconia on the Shear Bond Strength of Bilayered Porcelain/Zirconia

**DOI:** 10.3390/ma9090765

**Published:** 2016-09-08

**Authors:** Sebastian Spintzyk, Kikue Yamaguchi, Tomofumi Sawada, Christine Schille, Ernst Schweizer, Masahiko Ozeki, Jürgen Geis-Gerstorfer

**Affiliations:** 1 Section Medical Materials Science & Technology, University Hospital Tübingen, Osiander Strasse 2-8, Tübingen 72076, Germany; Sebastian.Spintzyk@med.uni-tuebingen.de (S.S.); christine.schille@med.uni-tuebingen.de (C.S.); ernst.schweizer@med.uni-tuebingen.de (E.S.); geis-gerstorfer@mwt-tuebingen.de (J.G.-G.); 2 Department of Implant Dentistry, Showa University School of Dentistry, 2-1-1 Kitasenzoku, Ota-ku, Tokyo 145-8515, Japan; kyamaguchi@dent.showa-u.ac.jp (K.Y.); ozekishowa@dent.showa-u.ac.jp (M.O.)

**Keywords:** yttria-stabilized tetragonal zirconia polycrystal (Y-TZP), veneering porcelain, shear bond strength, pre-sintered zirconia, surface modification

## Abstract

This study evaluated the bond strength of veneering porcelain with an experimental conditioner-coated zirconia. Pre-sintered Y-TZP specimens (*n* = 44) were divided in two groups based on conditioning type. After sintering, all sample surfaces were sandblasted and layered with veneering porcelain. Additionally, half of the specimens in each group underwent thermal cycling (10,000 cycles, 5–55 °C), and all shear bond strengths were measured. After testing, the failure mode of each fractured specimen was determined. Differences were tested by parametric and Fisher’s exact tests (α = 0.05). The differences in bond strength were not statistically significant. Adhesive fractures were dominantly observed for the non-thermal cycled specimens. After thermal cycling, the conditioner-coated group showed cohesive and mixed fractures (*p* = 0.0021), whereas the uncoated group showed more adhesive fractures (*p* = 0.0021). Conditioning of the pre-sintered Y-TZP did not change the shear bond strength of the veneering porcelain, but did improve the failure mode after thermal cycling.

## 1. Introduction

Commercial zirconia products are widely used not only as substrates of dental prostheses, but also as monolithic or full-contour restorations [1,2]. Based on a recent systematic review of bilayer ceramic restorations, zirconia-based single crowns have survival rates comparable with metal-ceramic single crowns [1]. Zirconia ceramics have superior mechanical properties that are similar to those of some metallic materials. Unfortunately, loss of retention and veneering ceramic fracturing are much more significant issues in zirconia-based ceramic restorations compared with metal-ceramic restorations [1].

To address the loss of retention issue, previous studies have indicated simple and clinically-reliable bonding methods in resin-bonded oxide ceramic restorations [3]. For ceramic fractures, complex mechanisms with a variety of risk factors are present in the oral cavity [4], making it difficult to identify the primary causes of chipping and delamination of veneering porcelain in clinical situations. However, sufficient bond strength between the veneering porcelain and the zirconia substructure is a key factor for the long-term clinical success of zirconia-based restorations [5].

For resolving failures of the veneering porcelain, various factors have been investigated in vitro using yttria-stabilized tetragonal zirconia polycrystal (Y-TZP) [6,7,8]. Sandblasting is the most widely-used surface treatment method of such systems, and can produce irregularities on zirconia surfaces to enhance the mechanical bonding [6]. Other factors (type of veneering material, veneering technique, number of firings, liner application, and cooling rate after sintering) can also influence the bond strength of the bilayered porcelain/zirconia [7,8]. In addition, some researchers have suggested that the framework design and thickness of the veneering porcelain must be considered [9,10]. Recently, alternative approaches including coating methods and plasma treatment for zirconia surfaces have been attempted for such studies [11,12].

At present, a number of complicated approaches have to be applied to zirconia surfaces to resolve these issues. However, the aforementioned factors can have either positive or negative effects on these systems. In particular, establishing the framework design is a technique-sensitive procedure that can easily influence the failure rates and fracture modes of the final restoration during manufacturing [3,13]. Furthermore, alternative approaches often require additional working time and/or special instrumentation. Consequently, it remains unclear how these approaches contribute to the bonding strength between the veneering porcelain and the zirconia surface during clinical use.

To establish a simplified and effective method for surface characterization, an alternative approach using an experimental slurry conditioner (a mixture of silicate ceramic and quartz) for promoting the adhesion between the veneering porcelain and the oxide ceramics is developed here [14]. This procedure can reduce the working time because this conditioner is simply applied on the pre-sintered zirconia surface prior to sintering. However, evidence of this conditioner’s impact on the bonding characteristics is inadequate at this time.

Thus, the purpose of this study is to test the shear bond strength between a veneering material and a commercial zirconia product treated with the conditioner before sintering, and evaluate the failure mode on its fractured surface. The null hypothesis was that the shear bond strength and failure mode in the conditioner-coated zirconia specimens would not improve compared with that of the uncoated zirconia specimens.

## 2. Materials and Methods

### 2.1. Materials

An experimental conditioner (Luxor Zirkonoxyd-Primer, Xplus 3, GmbH, Echzell, Germany) was obtained from the company [14]. The slurry conditioner consisted of a mixture of silicate ceramic and quartz, and contained SiO_2_ (74.1 wt %), Al_2_O_3_ (12.1 wt %) K_2_O (12.1 wt %), and Na_2_O (1.61 wt %) as the main components (>1 wt %) [15]. A commercial Y-TZP substrate (Nacera Pearl 1, Doceram, GmbH, Dortmund, Germany) and layering material (VM9, Vita Zahnfabrik, Bad Säckingen, Germany) were used.

### 2.2. Preparation of Zirconia Substrates

**Figure 1:**
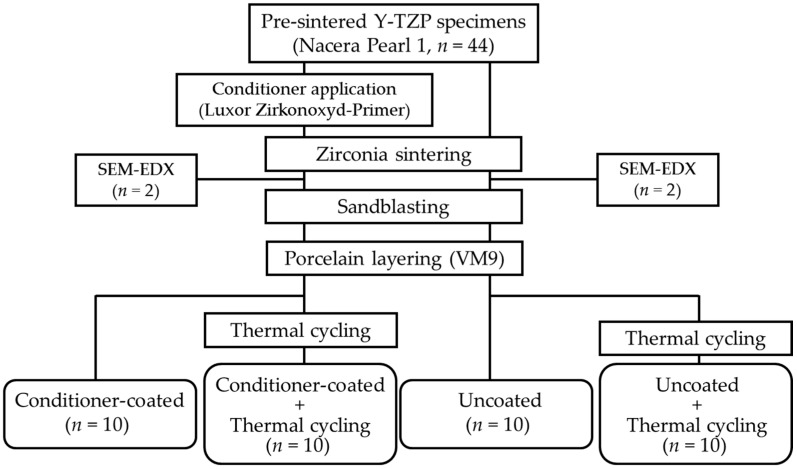
Experimental groups in this study.

Forty-four rectangular specimens (25 × 12 × 2.5 mm^3^) were milled out from pre-sintered Y-TZP blanks (Nacera Pearl 1, Doceram GmbH, Dortmund, Germany) using a CAD/CAM system (CORiTEC 450i, imes-icore GmbH, Eiterfeld, Germany). The surface of each specimen was polished with #320 and #1200 grit carbide papers (CarbiMet, Buehler, GmbH, Düsseldorf, Germany). All specimens were divided into two groups before sintering (Figure 1). Half of the specimens (*n* = 22) were pre-treated with the conditioner using a brush to obtain a thin conditioner layer on its surface. The rest of the specimens (*n* = 22) underwent no further treatment, and were used as a reference group. All specimens were sintered at 1380 °C for 2 h in a dental furnace (VITA ZYrcomat, VITA Zahnfabrik, GmbH, Bad Säckingen, Germany) (Table 1). After sintering, the conditioner-coated specimens (*n* = 2) were examined using scanning electron microscopy (SEM) along with energy dispersive X-ray spectroscopy (EDX) for elemental analysis at 10 kV. The surface of each specimen was sputter-coated with Au-Pd then observed using SEM (LEO 1430, Carl Zeiss AG, Oberkochen, Germany) to confirm the presence of the conditioner layer.

### 2.3. Porcelain Layering

**Figure 2:**
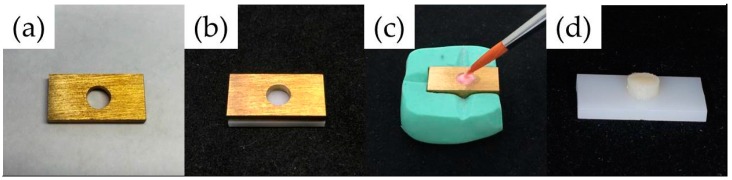
Procedure of porcelain layering: (**a**,**b**) a brass jig; (**c**) porcelain layering; and (**d**) the specimen after glazing.

Prior to porcelain layering, the rest specimens (20 × 10 × 1.9 mm^3^, *n* = 20 per group) were sandblasted with 120 µm Al_2_O_3_ particles (Spezial-Edelkorund, Harnisch + Rieth, GmbH, Winterbach, Germany) at 0.2 MPa for 20 s according to the manufacturer’s instructions and cleaned by a steam cleaner. Sandblasting was performed vertically at a fixed distance of 20 mm between the specimen surface and the nozzle using a special metal holder. Both conditioner-coated and uncoated specimens (mean roughness *R_a_*: 0.57 ± 0.12 μm and 0.40 ± 0.12 μm, respectively, measured using a contact profilometer, Perthometer SP6, Mahr GmbH, Göttingen, Germany) were veneered with feldspathic ceramic (VM9) using a layering technique. The porcelain layering procedure was comprised of five steps as follows: first and second wash-bake, first and second dentin, and glazing (Table 1). In order to standardize the layering process, each specimen was fixed using a brass jig (20 × 10 × 1.5 mm^3^) with a hole (5 mm diameter) corresponding to the center of the specimen (Figure 2a,b). The inner surface of the jig was coated with an isolating fluid (Carat, Hager, and Werken, Duisburg, Germany) to avoid adhesion of the veneering porcelain to the jig. Each porcelain powder was mixed with an appropriate amount of the respective liquid, and the mixture slurry was then filled into the jig (Figure 2c). After layering, the excess liquid was removed with tissue paper and the mold was carefully removed. Finally, each porcelain firing was performed in a dental furnace (Austromat 624, Dekema Dental-Keramiköfen, GmbH, Freilassing, Germany) according to the manufacturer’s instructions (Figure 2d).

Prior to testing the shear bond strength, half of the specimens in each group (*n* = 10) were subjected to thermal cycling for 10,000 cycles at alternating temperatures of 5 °C and 55 °C (70 s per cycle; dwelling time: 30 s, transfer time: 5 s) (Figure 1).

### 2.4. Shear Bond Strength Testing

**Figure 3:**
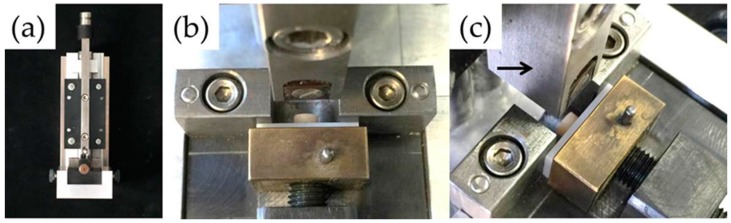
Shear bond strength testing: (**a**) a metal holder with a special designed metal piston; (**b**) fixation of a specimen; and (**c**) during shear bond strength testing.

Each specimen was fixed and placed in a metal holder which was connected with a universal testing machine (Z010, Zwick, GmbH, Ulm, Germany) (Figure 3a,b). Subsequently, the load was applied at the interface of each specimen using a special designed metal piston with a fixed carbide reversing plate (Figure 3c, black arrow) at a crosshead speed of 1.0 mm/min until debonding occurred according to ISO 10477 [16]. The shear bond strength was calculated in units of MPa using the maximum fracture load (N) divided by the bonding area (mm^2^) [17]. After testing, each fractured surface was analyzed by using stereomicroscopy (Wild Heerbrugg AG, Heerbrugg, Switzerland) or SEM. Failure modes were determined three different fracture patterns: adhesive, less than 33% of the porcelain remained on the zirconia surface; mixed, more than 33% but less than 66% of the porcelain remained on the zirconia surface (a combination of adhesive and cohesive fractures); and cohesive, more than 66% of the porcelain remained on the zirconia surface [18].

### 2.5. Data Analysis

Shear bond strength data were analyzed for normal distributions by the Shapiro-Wilks test and for variance equality by the Levene test. Bond strength results were then analyzed by a two-way analysis of variance (ANOVA) with conditioner application and thermal cycling as independent factors, followed by Tukey’s test for post-hoc comparisons. Descriptive statistics were applied using means and standard deviations. Failure mode results were analyzed by Fisher’s exact test with Benjamini-Hochberg multiple testing corrections [19]. Statistical analyses were performed by the software packages Excel Statistics 2010 (Social Survey Research Information Co., Ltd., Tokyo, Japan) and R version 3.2.3 (The R Foundation for Statistical Computing, Vienna, Austria) at a level of significance of *α* = 0.05.

## 3. Results

### 3.1. Surface Analysis

**Figure 4:**
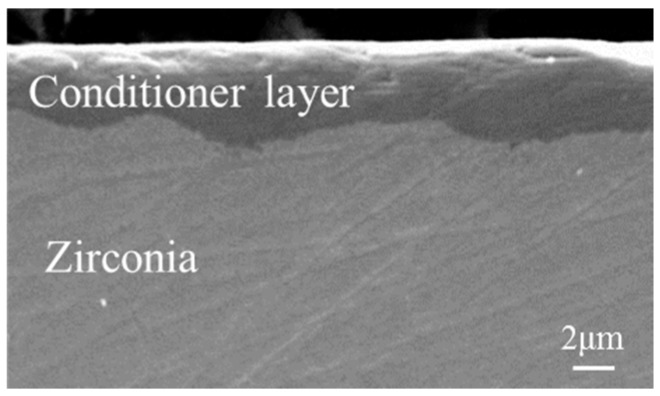
Cross-sectional scanning electron microscopy (SEM) micrograph of a conditioner-coated specimen (5000× magnification).

**Figure 5:**
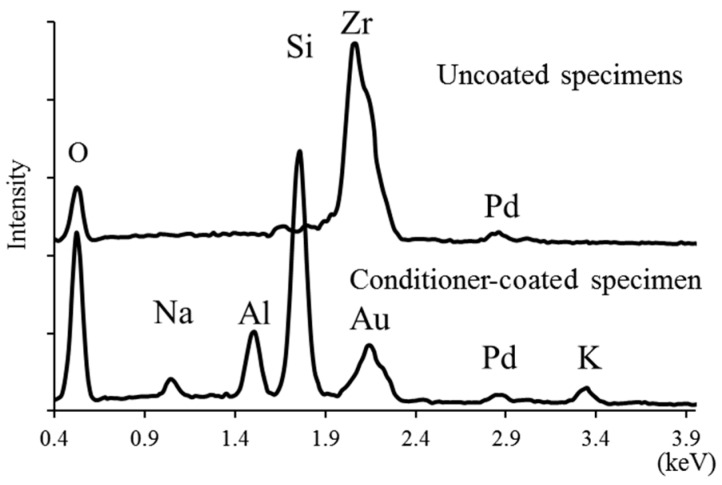
Energy dispersive X-ray spectroscopy (EDX) analyses of conditioner-coated and uncoated specimen surfaces.

SEM micrograph and EDX analysis of the conditioner-coated specimen after zirconia sintering are shown in Figure 4 and Figure 5, respectively. In the cross-sectional view, the whole surface was covered with a constant conditioner layer (approximately 5 µm) on the conditioner-coated specimen (Figure 4). The chemical elements of the conditioner-coated specimen surface were identified as sodium (Na), aluminum (Al), silicon (Si), potassium (K), and oxygen (O), whereas in the uncoated specimen only zirconia (Zr) and oxygen were identified (Figure 5). These findings confirmed the binding of the conditioner layer on the conditioner-coated specimen.

### 3.2. Shear Bond Strength

The shear bond strength data passed both the normality and equality tests for two-way ANOVA (*p* = 0.113 and 0.4007, respectively). The shear bond strengths ranged from 21.2 ± 5.8 MPa to 24.2 ± 5.2 MPa in these groups. The differences between the bond strengths of these groups were not significant, irrespective of thermal cycling (Table 2 and Table 3).

### 3.3. Analysis of Failure Modes

**Figure 6:**
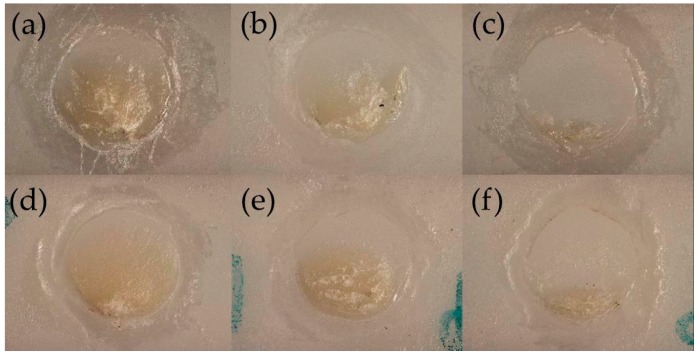
Microscope (10× magnification) of fractured specimen surfaces with different failure modes: (**a**) cohesive; (**b**) mixed; (**c**) adhesive failures in the uncoated group; (**d**) cohesive; (**e**) mixed; and (**f**) adhesive failures in the conditioner-coated group.

The distribution of different failure modes of each group is shown in Table 4. Without thermal cycling, adhesive fractures were dominantly observed in both conditioner-coated and uncoated specimens (Figure 6c), and there was no significant difference between the groups. Thermal cycling influenced the failure modes in different ways. Cohesive and mixed fractures (Figure 6d,e) were more common in the conditioner-coated group (*p* = 0.0021), whereas more adhesive fractures were observed in the uncoated group compared with the conditioner-coated group (*p* = 0.0021).

## 4. Discussion

This study focused on the influence of a specific conditioner method on the bonding of a zirconia surface with veneering porcelain. The shear bond strengths in the conditioner-coated specimens were not significantly different compared with those of the uncoated specimens, irrespective of thermal cycling. In the non-thermal cycled condition, adhesive fractures were dominantly observed in both types of specimens. On the other hand, after thermal cycling, cohesive and mixed fractures became more common in the conditioner-coated specimens, whereas higher numbers of adhesive fractures were observed in the uncoated specimens. Based on these findings, our null hypothesis was rejected.

The presence of the conditioner layer (approximately 5 µm) on the conditioner-coated specimens was confirmed by SEM-EDX (Figure 4 and Figure 5). Our approach using a conditioning method might be obtained, more or less, both chemical bonding and mechanical retention in the conditioner-coated specimens. One possible explanation for this behavior is that the experimental slurry conditioner infiltrated the pre-sintered zirconia surface and formed composite structures after sintering. According to a previous study [20], chemical bonding across the zirconia/glass ceramic interface was achieved by having thermodynamic stability as zirconia ions dissolve into the veneering porcelain. Thus, in this study, the chemical bonding between the Y-TZP surface and the conditioner layer might appear after sintering because the components of the conditioner are similar to those of the veneering porcelain. Moreover, mechanical retention was also obtained in the conditioner-coated specimens. Our assumption is supported by previous studies of synthetic, functionally-graded materials [21,22]. Qian et al. [21] formed graded glass/zirconia structures which were prepared by a coating glass-powder-containing slurry (different from our conditioner) onto Y-TZP surface before sintering. They explained that a certain degree of porosity existed in the pre-sintered zirconia, which could facilitate glass infiltration, a statement that is also reflected by our findings. 

The bonding characteristics between Y-TZP and the veneering porcelain could be explained by the interaction between mechanical retention and chemical bonding forces [23]. The cause of porcelain fracture is unknown in clinical situations but might be associated with bond failure between them [24]. To evaluate the bonding characteristics in this study, shear bond strength testing was used as a relatively simple method compared to microtensile bond strength testing [25]. Without thermal cycling, the conditioner-coated specimens showed similar initial shear bond strength compared to the uncoated specimens. Prior to porcelain layering, both the conditioner-coated and uncoated specimens were sandblasted. In general, surface roughening methods, such as sandblasting, are used to obtain mechanical retention [9]. In particular, sandblasting could improve interfacial adhesion by cleaning the zirconia surface or enhancing high surface energy and wettability [26]. Consequently, in dense Y-TZP, sandblasted substrates show higher shear bond strengths with veneering porcelain compared to polished substrates [27]. In contrast, some previous studies have indicated that sandblasting is not necessary to enhance bond strength, because strong chemical bonds are established between polished zirconia and veneering materials during firing [5,28]. Moreover, sandblasting may cause microfractures that would reduce the functional strength and lead to premature and catastrophic failure in zirconia [29]. These conflicting results are dependent on the zirconia substrates, the veneering materials, and the sandblasting conditions such as particle size, treatment time, and air pressure. Unfortunately, our conditioning method did not enhance the bond strength, but it could eliminate the negative influence on Y-TZP. 

Subsequently, the failure mode in the conditioner-coated specimens featured similar results, showing mainly adhesive failures compared to that in the uncoated specimens in the non-thermal cycled condition. Each failure mode was analyzed by measuring the amount of residual porcelain on the zirconia surface; only a small amount of porcelain remained on both experimental specimens. This was likely caused by the fact that surface modification did not improve the bond characteristics in both the uncoated and conditioner-coated specimens at this stage. This assumption may agree with previous studies of sandblasted zirconia materials, which were veneered with various porcelain materials and showed primary adhesive failures [25,30]. It is well known that the bond strength between the zirconia substrate and the veneering porcelain is the weakest link in layered structures [25]. Conversely, in another previous report, one sandblasted zirconia material that was veneered with seven different porcelain materials showed dominantly cohesive failures [31]. Furthermore, He et al. [27] showed dominantly mixed failure modes in the sandblasted specimens after sintering. These conflicting results were dependent on various factors. One possible explanation is that there is no clear and reliable evidence of failure mode classification for analyzing zirconia bonding characteristics to the veneering porcelain up to the present time. For instance, Guess et al. [17] analyzed the failure mode by measuring the residual veneering porcelain, like those examined in our study. When we applied our results to their classification, we identified a different result compared to this study. Thus, a variety of failure mode classifications exist and care should be taken when interpreting such data. In addition, even though zirconia specimens have rough surfaces after sandblasting, the failure modes depend on the sandblasting conditions. Sandblasting before zirconia sintering showed the shifting of failure modes, from mixed to cohesive, compared to that after zirconia sintering [27]. They explained that this difference was due to the fact that monoclinic phase of zirconia increased in volume after sandblasting, but this phase was not detected by reverse transformation; a monoclinic-to-tetragonal transformation may have occurred in the procedure of sandblasting before zirconia sintering. They also stated that an excessively rough surface might lead to stress concentration, which may consequently weaken the interfacial bonding between zirconia and porcelain. Thus, sandblasting conditions must be considered when discussing bond strength.

Thermal or/and mechanical cycling are used as conditions to simulate the artificial aging of zirconia bonding to the veneering porcelain [32,33]. In this study, in the thermal cycled condition, the conditioner-coated specimens showed similar shear bond strength compared to the uncoated specimens. These bond strengths were not significantly different compared to the initial bond strengths; our result is in agreement with previous studies. According to the aforementioned reports using different thermal conditions, bond strengths between different zirconia materials and veneering ceramics were not affected by thermal or/and mechanical cycling, and were evidently durable [17,32,33]. However, Y-TZP is susceptible to structural transformation from the tetragonal to the monoclinic phase under hydrothermal conditions (low-temperature degradation: LTD) [34]. Indeed, LTD is an aging phenomenon that occurs when the material is in contact with water [35]. One possible reason is that these thermal conditions were not long enough to cause a phase transformation because zirconia is a high rigid material. Thus, during this aging process, both specimens did not change in terms of shear bond strength.

By contrast, the distribution of failure modes differed noticeably. Thermal cycling promoted adhesive failures in the uncoated specimen whereas no adhesive failures were observed in the conditioner-coated specimens. This difference was due to the fact that thermal cycling may trigger the phase transformation at least in the uncoated specimens even though the fully-sintered Y-TZP has a lower volume fraction of pores compared to pre-sintered Y-TZP [36]. This conditioning method could protect from the surface flaws and microfractures after sandblasting due to the presence of the graded glass/zirconia structures in the conditioner-coated specimens, but small plastic deformation occurred in the uncoated specimens. In addition, thermal cycling might also influence and cause the degradation of the veneering porcelain. The risk of water contact in the conditioner-coated specimens might be lower compared to the uncoated specimens. Therefore, our assumption was that the weakest link was shifted from the interface between the conditioner and porcelain layers to the porcelain layer in the conditioner-coated specimens. Indeed, the synthetic, functionally-graded material architectures significantly reduced the stress concentration at interfaces and toughened all-ceramic restorations [19,37,38]. Therefore, in our study, the failure modes in the conditioner-coated specimens shifted from adhesive to mixed and cohesive after thermal cycling. Al-Dohan et al. [39] stated that adhesive failure did not occur in the presence of good bonding between compatible ceramic core and veneering materials. To summarize, conditioner-coated specimens did not negatively affect the shear bond strength to the veneering porcelain, but did enhance the failure mode after thermal cycling.

Alternative approaches of zirconia substrate modification have been reported by in vitro studies [40,41]. Porous zirconia, fabricated by adding pore-forming agents before sintering, showed strong shear bond strength against the veneering porcelain [40]. However, increasing the porosity of the zirconia leads to reduced flexural strength [40,41]. In contrast, our conditioner-coated specimens had less damage liability with regards to zirconia compared to the use of porous zirconia and zirconia sandblasted before sintering due to the presence of an intermediate layer.

Furthermore, in our previous report [15], the conditioner-coating zirconia with subsequent sandblasting inhibited the decrease of the shear bond strength to resin cement after thermal cycling, indicating improvements concerning the failure mode. Together with these results, the combination of conditioner application before sintering and sandblasting after sintering may improve both zirconia bonding situations simultaneously. Thus, this conditioning method is simple, safe, and economic compared with alternative treatments. However, we did not investigate the mechanical properties of the conditioner-coated zirconia. In addition, many types of commercial zirconia and veneering materials are present in the dental market [9,25]. These manufacturing procedures are different even though these materials share a similar chemical structure. Thus, for clinical use, further studies are needed to clarify the influence on the mechanical properties concerning this conditioner application on different zirconia surfaces.

## 5. Conclusions

Within the limitations of this in vitro study, the conclusions are as follows:

1. Conditioner-coated zirconia specimens did not show decreased shear bond strength compared with uncoated zirconia specimens, irrespective of thermal cycling;
2. In the analysis of fractured specimen surfaces, adhesive fractures were dominantly observed in both conditioner-coated and uncoated zirconia specimens without thermal cycling; and
3. In contrast, after thermal cycling the conditioner-coated zirconia specimens predominantly showed cohesive and mixed fractures (*p* = 0.0021), whereas uncoated zirconia specimens showed higher numbers of adhesive fractures (*p* = 0.0021).

Thus, this conditioner method on pre-sintered Y-TZP did not change the shear bond strength of the veneering porcelain, but led to improved failure modes after thermal cycling.

## Figures and Tables

**Table 1:** Zirconia sintering and porcelain firing schedules.

Procedure	Basic Temperature (°C)	Dry Time (min)	Temperature Increase (°C/min)	High Temperature (°C)	Hold Time (min)
Y-TZP sintering	20	-	8	1380	120
Porcelain firing					
1st/2nd wash-bake	500	6	55	930	1
1st dentin	500	6	55	910	1
2nd dentin	500	6	55	900	1
Glaze	500	4	80	900	1

**Table 2:** Two-way analysis of variance (ANOVA) of shear bond strength.

Source	DF	Sum of Squares	Mean Square	F	*p*
Conditioner application (A)	1	0.0363	0.0363	0.0013	0.9714
Thermal cycling (B)	1	64.8929	64.8929	2.3259	0.1360
(A) × (B)	1	1.7189	1.7189	0.0616	0.8054
Error	36	1004.4042	27.9001		
Total	39	1071.0523			

**Table 3:** Mean (standard deviation) shear bond strength value of each group in MPa.

Group	Shear Bond Strength
Uncoated	23.7 (4.1) ^A^
Conditioner-coated	24.2 (5.2) ^A^
Uncoated + Thermal cycling	21.6 (5.9) ^A^
Conditioner-coated + Thermal cycling	21.2 (5.8) ^A^

Results of statistical analysis are represented by upper case letters. Same uppercase letters mean that the groups are not significant different (*p* < 0.05).

**Table 4:** Numbers of different failure modes of each group.

Group	Cohesive	Mixed	Adhesive	*p*
Uncoated	3	1	6	A,B
Conditioner-coated	1	1	8	A
Uncoated + Thermal cycling	1	1	8	A
Conditioner-coated + Thermal cycling	4	6	0	B

Results of statistical analysis are represented by upper case letters. Same uppercase letters mean that the groups are not significantly different (*p* < 0.05).
